# Distribution and Dynamic Habitat Use of Young Bull Sharks *Carcharhinus leucas* in a Highly Stratified Northern Gulf of Mexico Estuary

**DOI:** 10.1371/journal.pone.0097124

**Published:** 2014-05-19

**Authors:** J. Marcus Drymon, Matthew J. Ajemian, Sean P. Powers

**Affiliations:** 1 Department of Marine Sciences, University of South Alabama, Mobile, Alabama, United States of America; 2 Center for Ecosystem Based Fishery Management, Dauphin Island Sea Lab, Dauphin Island, Alabama, United States of America; Aristotle University of Thessaloniki, Greece

## Abstract

Understanding how animals alter habitat use in response to changing abiotic conditions is important for effective conservation management. For bull sharks (*Carcharhinus leucas*), habitat use has been widely examined in the eastern and western Gulf of Mexico; however, knowledge of their movements and the factors influencing them is lacking for populations in the more temperate north-central Gulf of Mexico. To examine how changes in hydrographic conditions affected the presence of young bull sharks in Mobile Bay, Alabama, thirty-five sharks were fitted with internal acoustic transmitters and monitored with an acoustic monitoring array consisting of thirty-three receivers between June 2009 and December 2010. Tagged sharks ranged in size from 60 to 114 cm fork length and were detected between the upper and lower portions of Mobile Bay. Despite a variety of freshwater sources associated with this highly productive estuary, sharks were most consistently detected at the largest input to the system – the Mobile and Tensaw Rivers. Our findings suggest a combination of hydrographic factors interact to influence the distribution of juvenile bull sharks in Mobile Bay. The factors affecting the probability of detecting at least one bull shark varied both temporally (2009 vs 2010) and spatially (upper vs lower bay). Electivity analysis demonstrated that bull sharks showed highest affinity for warm water (29–32°C), moderate salinities (10–11 psu) and normoxic waters (5–7 mg/l), although these patterns were not consistent between regions or across years. We suggest future studies coupling telemetry and hydrographic variables should, when possible, consider the interactions of multiple environmental parameters when defining the dynamic factors explaining the spatial distribution of coastal sharks.

## Introduction

Coastal ecosystems are composed of a suite of important habitats for many shark species. The habitats within these ecosystems can serve as nursery areas for young-of-the-year and juvenile sharks [1], [2] and foraging habitats for all life stages [3]. The use of coastal habitat is influenced by a range of biotic and abiotic factors [4], [5]. Biotic factors include prey abundance [6], as well as reduced predation risk leading to decreased mortality [7]. Abiotic factors influencing the use of coastal habitat by sharks include temperature [8], salinity [9], [10], and dissolved oxygen [11]. Given the increased urbanization of coastal areas, advancing our understanding of the mechanisms influencing habitat use by sharks can provide predictive capability in the face of changing environmental conditions.

Bull sharks (*Carcharhinus leucas*) are known to inhabit coastal areas circumglobally along warm temperate to subtropical clines. As one of only a few euryhaline sharks [12], bull sharks are able to tolerate significant abiotic fluctuations associated with dynamic coastal habitats and have thus been the focus of several studies within these areas. Using survey data, previous studies from the US have identified the importance of Matagorda Bay, Texas [2] and the Indian River Lagoon, Florida [13] as central nursery areas for bull sharks. Catch data has also illustrated the importance of temperature and salinity in determining habitat use for juvenile bull sharks [14], [15]. These relationships have been further supported through acoustic telemetry, including active tracking [16], [17] and passive monitoring. Acoustic monitoring arrays in southwest Florida have been used to document bull shark habitat use in Pine Island Sound [18] and the Caloosahatchee River estuary [19], [20]. While juvenile bull shark habitat use has been well studied in the western and eastern Gulf of Mexico, knowledge of their movements and the factors influencing them is less abundant for populations in the more temperate north-central Gulf of Mexico. A synthesis of available datasets from coastal Louisiana demonstrates neonate and juvenile bull shark occurrence throughout saline, brackish and freshwater environments [21]. Similarly, the presence of neonate and juvenile bull sharks has been documented through fishery-independent gillnet sampling in the coastal waters of Mississippi and Alabama [22]. The north-central Gulf of Mexico, inclusive of coastal Louisiana and the Mississippi Bight, is best classified as river dominated. In contrast to the eastern and western extremes of the Gulf of Mexico, dramatic intra and inter-annual variation related to freshwater input dominates the abiotic regime and may be related to climatic oscillations on longer time scales [23]. Understanding habitat use and population dynamics under these conditions is critical to developing a more complete understanding of the ecology of coastal sharks in this region.

As anthropogenic influences continue to alter coastal marine environments, particularly freshwater inputs, habitats that were once suitable may become uninhabitable for estuarine sharks. Our current understanding of habitat use by immature bull sharks is based largely on acoustic tagging studies from south Florida estuaries. Estuaries in the north-central Gulf of Mexico are more dynamic systems, subject to colder temperatures and wider ranges in salinity. Thus, investigating how bull sharks use such estuarine systems is critical if we are to better predict how anthropogenic alterations may affect the spatial distribution of sharks in these different regions. The objectives of this study were to examine spatial patterns in habitat use and describe the relationship between abiotic variables and the distribution of juvenile bull sharks in Mobile Bay, Alabama, monitored with passive acoustic telemetry.

## Methods

### Ethics Statement

This study was conducted in accordance with the laws of the state of Alabama and under the IACUC protocols (IACUC Board Reference Number 11014) approved by the University of South Alabama. All sampling occurred in state waters under permits granted by the State of Alabama Department of Conservation and Natural Resources Marine Resource Division to the authors. Sharks were collected as part of ongoing, standardized surveys (see *Acoustic Tagging* section below). All efforts were made to reduce animal suffering during handling and tagging procedures.

### Study Site

Field sampling occurred in Mobile Bay, Alabama, a dynamic, drowned valley estuary in the north-central Gulf of Mexico. The bay is large and shallow, extending ∼50 km north to south and 14–34 km east to west with a mean depth of approximately 3 m. Mobile Bay receives the second largest river discharge of all estuaries in the Gulf of Mexico, primarily from the Alabama and Tombigbee rivers, resulting in large seasonal fluctuations in abiotic parameters [24].

### Acoustic Array and Range Testing

**Figure 1:**
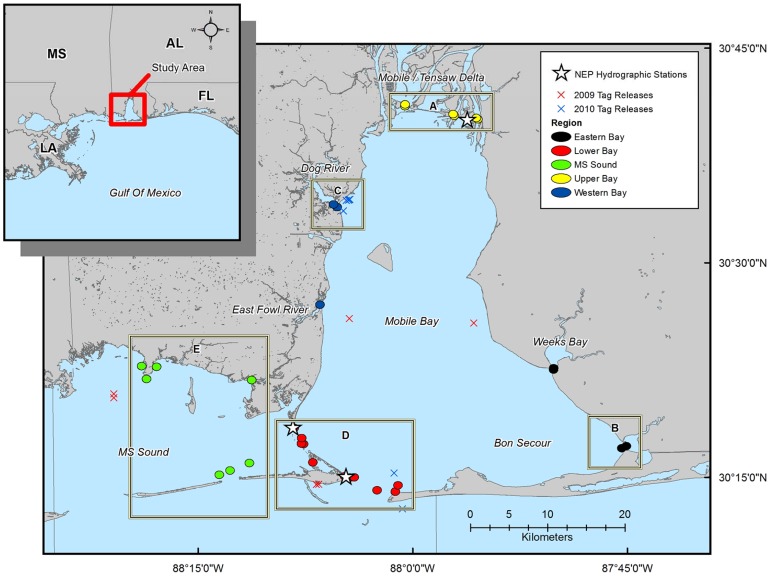
Map of the Coastal Alabama Acoustic Monitoring Program (CAAMP). The circles indicating receiver locations are scaled to the approximate mean detection range, and are coded by region: Upper Bay in yellow (A, n = 6), Eastern Bay in black (B, n = 4), Western Bay in blue (C, n = 5), Lower Bay in red (D, n = 11), and MS Sound in green (E, n = 7). The white stars indicate the location of the National Estuary Program (NEP) mooring stations from which hydrographic data were obtained. X's indicate release locations for the telemetered bull sharks in 2009 (red) and 2010 (blue) subsequently detected in the array.

Thirty-three Lotek Wireless Hydrophone System (WHS) omni-directional acoustic receivers were deployed in Mobile Bay as part of the Coastal Alabama Acoustic Monitoring Program (CAAMP, Figure 1). The CAAMP array employed two receiver models: Lotek WHS2000s (n = 27) and WHS3050s (n = 6). These receivers detected two separate types of acoustic coding technology; the WHS3050 receivers detected MAP code transmissions, and the WHS2000 receivers detected Rcode transmissions (see transmitter specifications below). Receiver attachment method varied according to location. The majority of receivers were attached to bottom moorings (n = 21), although some were attached to dock pilings (n = 6) or channel markers (n = 6) in regions of high vessel traffic and strong currents. To monitor ingress and egress from Mobile Bay, we placed hydrophones in a “curtain” array at freshwater inputs (Mobile-Tensaw Delta, Dog River, Weeks Bay, etc,) along the periphery of the study area. This array design was chosen based on the objectives of our study, and the cost associated with deploying and maintaining a gridded array across the entire bay [25]. All receivers were present throughout the course of the study area and recorded time, date and the identity of tagged animals that passed within the detection range of the receiver.

Range testing was conducted six times between March and October 2010 to monitor receiver performance over a wide range of hydrographic conditions. Range test tags (same model as those applied to animals; see below) were lowered over the side of a boat (i.e. boat-based method) [26] at varying intervals from the acoustic receiver 1 m from the bottom. At each interval the number of detections per five minute period was recorded. All range testing was conducted with shallow water receivers moored between 2 and 3 m deep, free from surface noise and turbulence. The vessel outboard engine was turned off during testing and the boat was anchored in place. In addition to range testing, acoustic receivers were serviced every 2 months, during which time data were downloaded, biofouling removed, and batteries replaced (as needed).

### Specimen Capture and Acoustic Tagging

Sharks were captured with gillnets and longlines as part of ongoing standardized abundance surveys of elasmobranch assemblages in the Mobile Bay estuary [27], [28]. Once captured, juvenile bull sharks were removed, measured (pre caudal length, fork length and stretched total length in mm), weighed (to nearest 0.1 kg) and tagged. Sharks were fitted with two tag types; an external, plastic swivel tag (Dalton ID, Henely-on-Thames, UK) and an internal ID only acoustic tag (Lotek model MM-MR-16-50, 16×80 mm, 35 g in air). The MM-MR-16-50 is a multi-mode transmitter, set to pulse every 5 seconds on a MAP code frequency (76.8 kHz, detected by the WHS3050) and every 60 seconds on a an Rcode frequency (69 kHz, detected by the WHS2000). For internal tags, a small incision was made above midline of the ventral surface for implantation. Tags were surgically inserted into the peritoneal cavity, and the incision was closed with surgical sutures (3.0 Ethicon Prolene monofilament). Once closed, an antiseptic wipe was applied to the sutured area. The time needed to apply both the external swivel and the internal acoustic tags ranged from 90–360 seconds.

### Hydrographic Datasets

To relate the presence of telemetered bull sharks to abiotic parameters, hydrographic data were obtained from mooring stations maintained by the Dauphin Island Sea Lab (DISL) and the Mobile Bay National Estuary Program (MBNEP) (Figure 1). At each station temperature, salinity and dissolved oxygen data were measured with a YSI 6600 (Yellow Springs Instruments, Inc.) every 30 minutes. These data are displayed in near real-time, and were available for download at http://www.mymobilebay.com. Data were available for every day of the year; for our analysis, we downloaded data from 2009 and 2010, and reduced those data to the range of dates hydrophones were actually deployed and tags were actively transmitting within the array.

### Data Analysis

All data were analyzed in XLstat version 13.0 (Addinsoft, Inc.). Detection data from the acoustic receivers were used in conjunction with environmental data from the hydrographic mooring stations to examine potential factors influencing bull shark presence within the acoustic array. For the acoustic receivers, bull sharks were considered present near a hydrophone if >1 detection occurred within a thirty minute period. From these data, presence plots were constructed. To link the acoustic detection data to the hydrographic data, bull shark detections were collated into thirty minute bins and linked to the hydrographic data using the time stamp. Bull shark presence/absence, temperature, salinity and dissolved oxygen data were synchronized every thirty minutes throughout the time bull sharks were detected within the acoustic array. Boxplots and dynamic habitat use plots [29] were used to visualize the relationship between the presence of telemetered bull sharks and single and combinations of hydrographic variables, respectively. A generalized linear model with a binomial probability distribution and a logit link was used to determine if the predictor variables temperature, salinity, dissolved oxygen or their combinations could predict the probability of detecting at least one bull shark. Firth's penalized maximum likelihood method [30] was used to reduce the bias associated with the small (relative) proportion of positive detections in comparison to large periods of absence. The significance of the regression coefficients for individual predictors was evaluated using the Wald statistic and overall performance of the model was assessed by examining the receiver operating curves (ROC). Values of ROC range from 0.5 to 1, where 1 suggests perfect discrimination between presence and absence probabilities, and 0.5 suggests the model performance is no better than random. Values greater than 0.8 are considered very good, and values above 0.9 excellent [15]. Given both the wide spatial variation in abiotic data, and the difference in detection probability resulting from unequal acoustic receiver coverage throughout Mobile Bay, we separated the bay into upper and lower components. In addition, since the hydrophone array was deployed for less time in 2009 than 2010, we separated the two years of acoustic monitoring data. A total of 4 models were constructed: lower bay 2009, lower bay 2010, upper bay 2009, and upper bay 2010. Electivity values were calculated for both regions (lower and upper bay) and for both years (2009 and 2010) following Chesson [31] to determine if telemetered bull sharks displayed dynamic habitat use ranging from avoidance (0) to affinity (1) for various abiotic regimes. Since hydrographic conditions varied between years, neutral values for Chesson's alpha were standardized by subtracting the value 1/(number of categories) following Heupel and Simpfendorfer [19].

## Results

### Range tests

**Figure 2:**
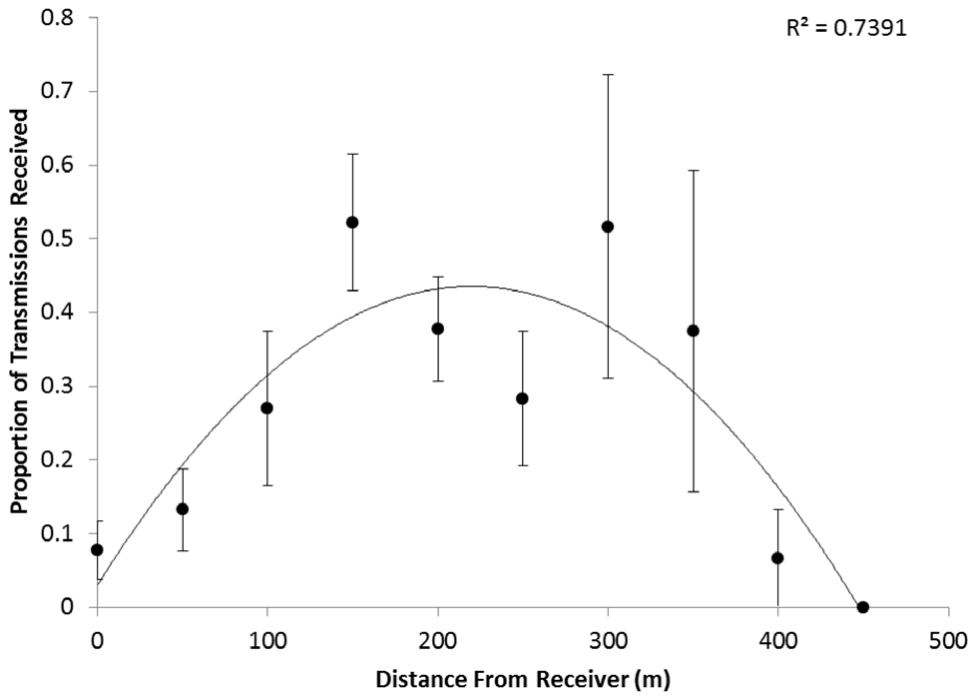
Results of boat-based range testing the WHS 2000 acoustic receivers. Mean proportion of transmissions detected are shown as a function of distance from the receiver, in meters. Error bars are SE.

Analysis of the range test data showed the two receiver types we used had different optimal detection ranges. The vast majority (>80%) of the CAAMP array was composed of WHS2000 receivers. On these receivers, acoustic tags were detected at distances ranging from 0 to 400 meters from the receiver. Optimal detection (the highest proportion of detections) occurred at 150 and 300 m from the receivers, although the overall detection pattern was well described by a second order polynomial fit (R^2^ = 0.74) (Figure 2). In contrast, range testing of the few WHS3050 receivers used in the array showed a markedly different detection pattern. Acoustic tags were detected between 0 and 300 meters, yet the proportion of tags detected was strongest at the receiver, and decreased with increasing distance from the receiver, a trend best described by an exponential decay (R^2^ = 0.92).

### Acoustic tagging

**Figure 3:**
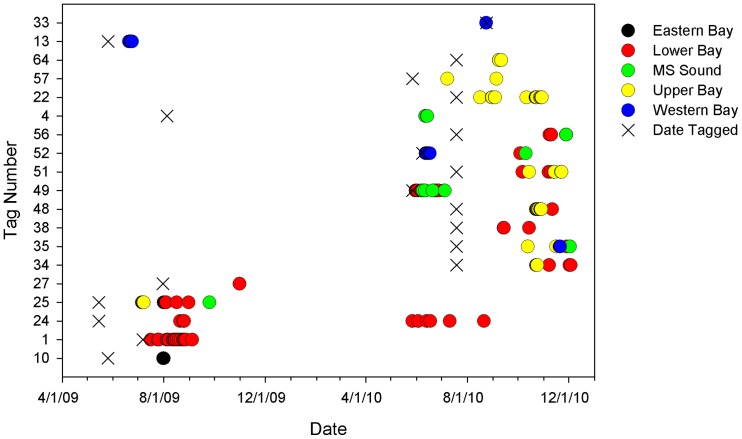
Presence history for acoustically tagged bull sharks. Data are shown for sharks detected in 2009 and 2010, coded by region. The gray area indicates the period when acoustic receivers were not deployed. X's mark the dates tags were deployed on sharks.

**Figure 4:**
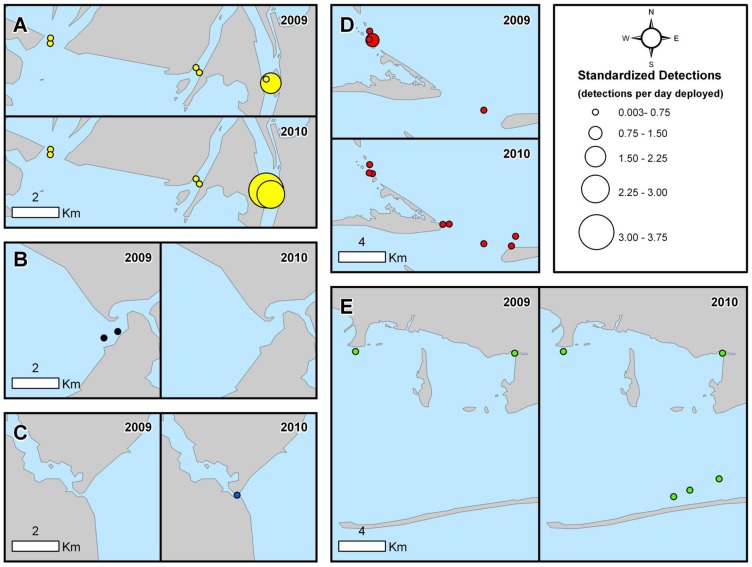
Distribution of bull shark detections. Number of bull shark acoustic detections (detections per day deployed) throughout the CAAMP array in 2009 and 2010. Detections were standardized both to the receiver type (WHS 3050 vs WHS 2000, which had different detection intervals) and the number of days receivers were deployed. Colors (yellow, red, blue, black, and green) and letters (A–E) are consistent with Figure 1.

Between May 2009 and August 2010, thirty-five bull sharks were fitted with internal acoustic transmitters. Nineteen of these fish were subsequently detected in our acoustic array between June 2009 and December 2010 (Figure 3). Sharks acoustically tagged in this study were relatively small, ranging in size from 60 to 114 cm fork length (FL; Table 1), with a median size of 68 cm FL and a mean size of 77 cm FL (±4.07 cm SE). Collectively, this size range is indicative of individuals that are primarily neonate and young-of-the-year [32]. Interestingly, one of the larger sharks (ID number 4) tagged in the summer of 2009 was not detected in the array until the following summer (Table 1). Spatial patterns in acoustic detections were mapped to examine the primary regions of activity within the CAAMP array (Figure 4), and showed that the acoustic receivers in the upper and lower portions of Mobile Bay recorded the most detections (91.4%). In particular, the northeast portion of Mobile Bay appeared to be a “hotspot” for tagged bull sharks, with more relative detections in that area than any other region in the array (Figure 4A). Given the low number of detections throughout Mississippi Sound and the western and eastern portions of Mobile Bay, the remaining analyses focused on the upper and lower regions of Mobile Bay.

**Table 1:** Biological data for tagged bull sharks.

ID	FL (cm)	Sex	Date tagged
24	74	F	5/15/09
25	110	M	5/15/09
13	60	M	5/26/09
10	64	M	5/26/09
1	96	M	7/8/09
27	68	F	7/31/09
4	95	F	8/5/09
49	106	M	5/27/10
57	76	F	5/27/10
52	80	F	6/8/10
22	67	F	7/19/10
34	61	M	7/19/10
35	60	M	7/19/10
38	67	F	7/19/10
48	65	F	7/19/10
51	69	F	7/19/10
56	68	F	7/19/10
64	67	F	7/19/10
33	690	M	8/23/10

Biological data (length, cm FL and weight in kg) for bull sharks acoustically tagged in 2009 and 2010.

### Hydrographic data

**Figure 5:**
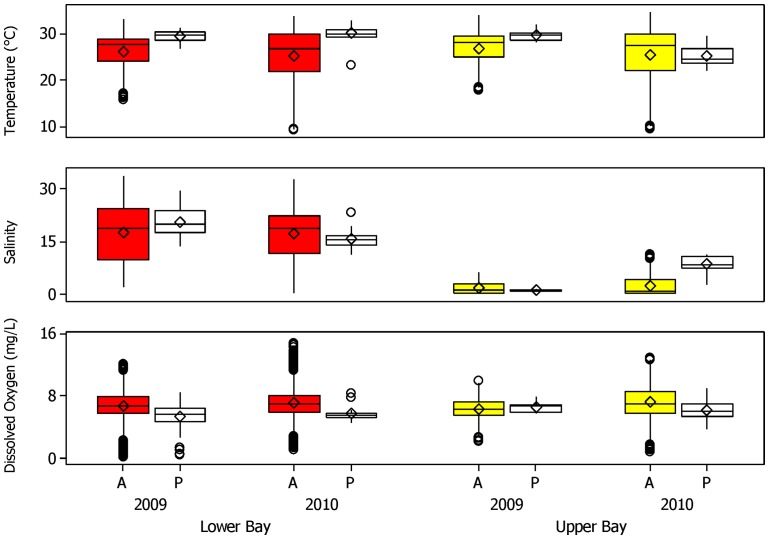
Bull shark presence as a function of hydrographic data. Box and whisker plots (median, interquartile range) of temperature, salinity and dissolved oxygen are shown during periods of telemetered bull shark absence (A) and presence (P) in the lower (red) and upper (yellow) portions of Mobile Bay. Mean values are shown with diamonds, and circles indicate outliers.

Temperature, salinity and dissolved oxygen values were recorded during the study periods in 2009 (July – November) and 2010 (March – December) in the upper and lower portions of Mobile Bay. Between these two regions, mean temperature varied little, with values of 26.15°C (±0.04 SE) and 26.98°C (±0.05 SE) in the upper and lower bay in 2009. A similar trend, yet with slightly lower values was seen in 2010 with mean temperatures of 25.38°C (±0.04 SE) and 25.41°C (±0.05 SE) in the lower and upper bay, respectively. Similarly, dissolved oxygen values showed little difference between region, where mean DO was 6.78 mg/l (±0.02 SE) and 6.34 mg/l (±0.02 SE) in the lower and upper bay, respectively. As was the case with temperature, dissolved oxygen values in both regions were slightly higher in 2010, where mean DO was 7.09 mg/l (±0.01 SE) and 7.20 mg/l (±0.02 SE) in the upper and lower bay, respectively. As expected, salinity values showed the most marked differences between regions, as well as between years. In 2009, mean salinity values in the lower and upper regions were 17.58 psu (±0.08 SE) and 1.52 psu (±0.02 SE), respectively. In 2009, the range of salinity values in the lower bay was 31.87 and 6.02 in the upper bay. In 2010, the salinity regime in the lower bay was similar to the previous year, with mean salinity  = 17.18 psu (±0.05 SE) and a range of values of 32.98. In the upper bay, mean salinity was slightly higher in 2010 compared to 2009, with a mean value of 2.31 psu (±0.03 SE); however, a much larger range of salinity (11.26) was recorded in 2010 relative to 2009 (Figure 5).

### Relating telemetered sharks to hydrographic variables

Bull sharks acoustically monitored in the lower and upper portions of Mobile Bay were not detected across the entire range of hydrographic conditions. In the lower bay, bull sharks were only detected in relatively warm waters, where they were present across 25% of the available range of temperatures in 2009. In 2010 the range of temperatures that tagged bull sharks were detected in increased to nearly 40% of the available temperatures, although this increase was due to the detection of a single individual. With respect to DO, acoustically tagged sharks in the lower bay were detected across two thirds of the available values of DO in 2009, yet only across 28% of the available DO regimes in 2010. Acoustically tagged bull sharks in this study were detected across nearly half of the available salinity values in 2009, decreasing to approximately 40% in 2010 (Figure 5). In 2009, tagged bull sharks in the upper bay were detected towards the upper portion of the available thermal conditions, detected across nearly a quarter (23%) of the available temperature values. This increased slightly in 2010 (29%), similar to what was seen with respect to temperature in the lower bay. For dissolved oxygen, the pattern was similar, with bull sharks detected at over a quarter (27%) of the available DO values in 2009 in the upper bay, increasing to 43% in 2010. The most striking difference between the available hydrographic regimes and the ones where bull sharks were detected was with respect to salinity. In 2009, tagged bull sharks were detected at only 13% of the available salinity values, where the mean value of salinity when bull sharks were present  = 0.95 psu (±0.04 SE). In contrast, tagged bull sharks were detected across greater than three fourths (77%) of the available salinity values in 2010, where the mean value of salinity when bull sharks were present was 8.43 psu (±0.18 SE) (Figure 5).

**Figure 6:**
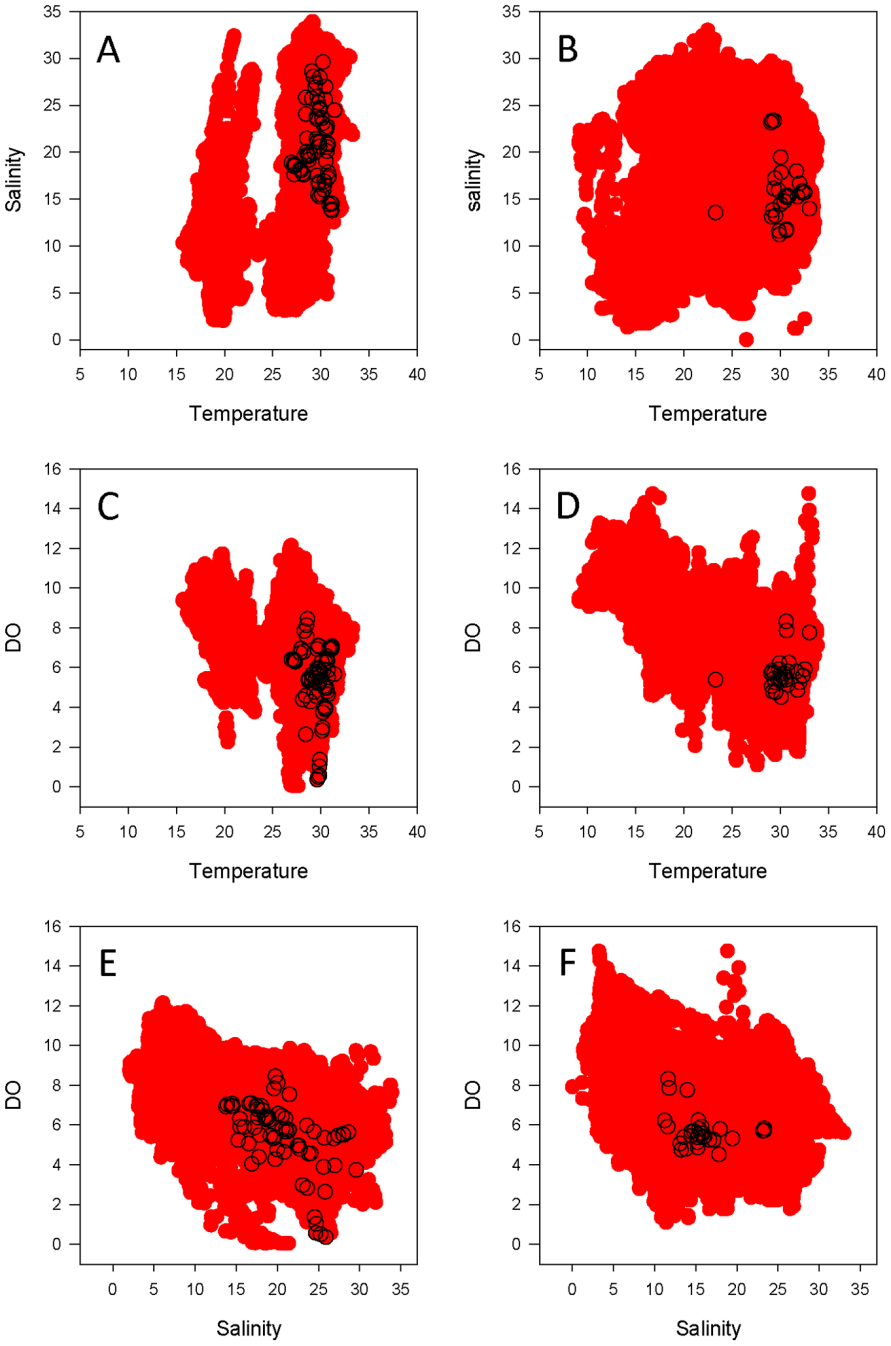
Bull shark dynamic habitat use in the lower bay. Dynamic habitat use of the lower Mobile Bay by bull sharks acoustically detected in 2009 (left column) and 2010 (right column): Temperature vs. Salinity (A, B), Temperature vs. Dissolved Oxygen (C, D), and Salinity vs. Dissolved Oxygen (E, F) are shown. Areas in red indicate the available dynamic habitat (data from the NEP mooring stations), and open circles indicate the presence of telemetered bull sharks.

**Figure 7:**
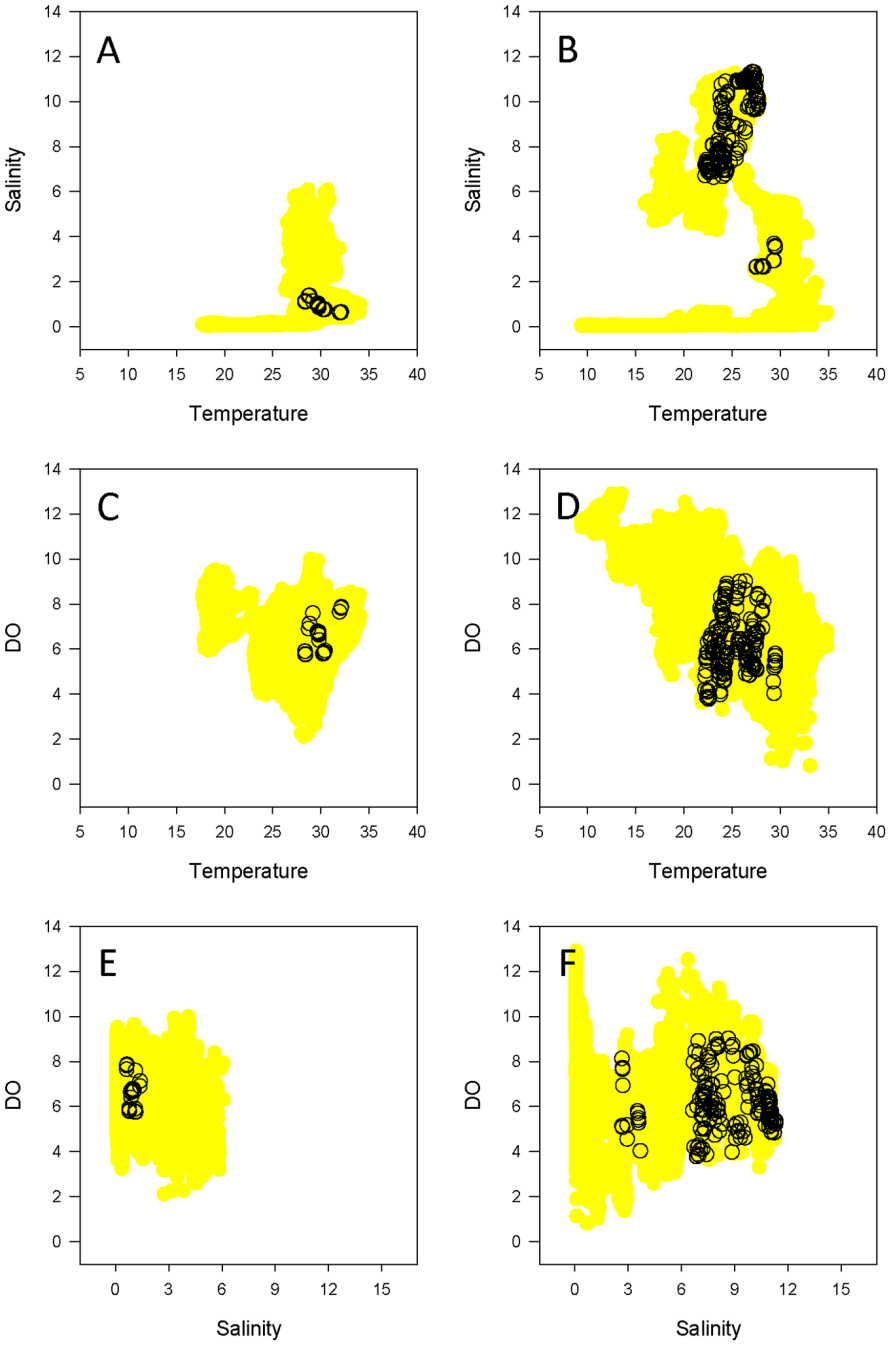
Bull shark dynamic habitat use in the upper bay. Dynamic habitat use of the upper Mobile Bay by bull sharks acoustically detected in 2009 (left column) and 2010 (right column): Temperature vs. Salinity (A, B), Temperature vs. Dissolved Oxygen (C, D), and Salinity vs. Dissolved Oxygen (E, F) are shown. Areas in yellow indicate the available dynamic habitat (data from the NEP mooring stations), and open circles indicate the presence of telemetered bull sharks.

Dynamic habitat use varied as a function of both region and year. In 2009, bull sharks monitored in the lower bay used a relatively narrow range of temperature in conjunction with a wider range of salinity and DO (Figure 6A and C). A similar pattern was observed in 2010 (Figure 6B and D). Whereas the prevailing hydrographic regimes in the lower bay were similar in 2009 and 2010, a higher degree of annual variability was seen in the upper bay, particularly with respect to salinity. The upper bay experienced a much narrower range of salinity in 2009 (Figure 7A and E) compared to 2010 (Figure 7B and F), such that the salinity occupied by bull sharks in 2010 did not occur in the upper bay in 2009. Conversely, ambient temperature values were more similar between years, as were the temperatures occupied by telemetered bull sharks (Figure 7A–D).

Maximization of Firth's penalized likelihood function identified different combinations of the predictor variables across both region and year. In general, the models showed poorer fit in 2009 compared to 2010. In 2009, the best fit model for the lower bay included the terms temperature, DO, temperature*salinity, temperature*DO and salinity*DO. The ROC value of 0.847 indicated very good model discrimination. Standardized regression coefficients indicated that temperature (p<0.01), DO (p<0.01) and their interaction (p<0.01) were the most influential parameters for the lower bay model in 2009. In 2010, the best fit model for the lower bay included the terms temperature, temperature*salinity, and temperature*DO. The ROC value of 0.864 indicated very good model discrimination. Standardized regression coefficients indicated that temperature (p<0.01), temperature*salinity (p<0.01) and temperature*DO (p<0.01) were equally influential parameters for the lower bay model in 2010. In the upper bay model for 2009, the best fit model included the terms temperature, DO, and temperature*salinity. The ROC value of 0.817 indicated a well-discriminated model. Standardized regression coefficients indicated that temperature (p<0.01) and the temperature*salinity interaction (p<0.01) were the most influential parameters in the upper bay model for 2009. In 2010, the best fit model included the terms temperature, salinity and DO, as well as all the first order interactions. The ROC value of 0.937 indicated excellent model discrimination. Standardized regression coefficients indicated that all predictors (p<0.01) were equally influential parameters for the upper bay model in 2010 (Table 2).

**Table 2:** Summary of predictive model results.

Model	Factor	p value
**2009 Lower Bay**	*Temperature*	<0.001
AIC = 706.524	*DO*	<0.001
ROC = 0.847	Temp*Salinity	0.02
	Temp*DO	<0.001
	Salinity*DO	0.111
**2010 Lower Bay**	*Temperature*	<0.001
AIC = 412.876	Temp*Salinity	<0.001
ROC = 0.864	Temp*DO	0.001
**2009 Upper Bay**	*Temperature*	<0.001
AIC = 281.561	DO	0.884
ROC = 0.817	Temp*Salinity	0.005
**2010 Upper Bay**	*Temperature*	<0.001
AIC = 1113.765	*Salinity*	<0.001
ROC = 0.937	*DO*	<0.001
	Temp*Salinity	<0.001
	Temp*DO	<0.001
	Salinity*DO	0.005

Summary of GLM results for predicting the probability of detecting at least one bull shark as a function of region (upper bay and lower bay) and year (2009 and 2010). Significant values (α<0.005) are indicated in italics.

**Figure 8:**
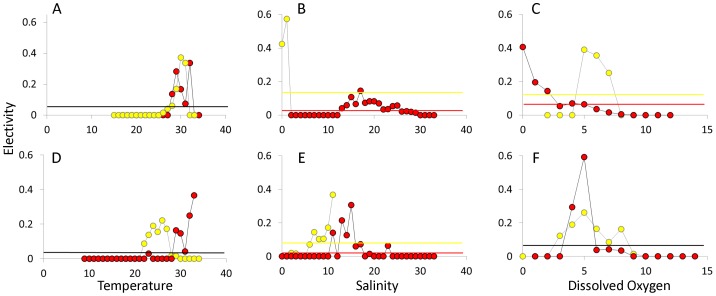
Bull shark electivity. Electivity plots for the lower (red) and upper (yellow) portions of Mobile Bay in 2009 (A–C) and 2010 (D–F). Electivity data are plotted for temperature (A, D) salinity (B, E) and dissolved oxygen (C, F). Horizontal lines represent neutral selection in the upper (yellow) and lower (red) bay. Horizontal black lines are used when the neutral selection value is the same between the upper and lower bay.

Electivity analyses were used to further investigate whether bull sharks showed an affinity for or avoidance of a specific range of hydrographic variables. In 2009, temperature electivity values were similar between the lower and upper bay regions, with affinity demonstrated for temperatures between 29 and 32°C. In 2010, the pattern in the lower bay was similar to the previous year, but bull sharks showed affinity for cooler temperatures (24–26°C) in the upper bay compared to 2009 (Figure 8A & D). With respect to salinity, in 2009 bull sharks demonstrated strong affinity for salinity regimes between 0 and 1 in the upper bay, with no selection for salinity regimes in the lower bay. In 2010, strong affinity was shown for salinity values of 10 and 11 in the upper bay, similar to what was seen in the lower bay (Figure 8B & E). In the lower bay in 2009, affinity for low DO waters was observed, where electivity increased with decreasing DO, reaching peak electivity when waters were hypoxic (0–1 mg/l). In contrast, telemetered bull sharks in the upper bay in 2009 showed strongest affinity for normoxic waters (5–7 mg/l). In comparison, bull sharks monitored in the lower bay in 2010 showed affinity for normoxic waters. A similar, but less pronounced pattern was seen with respect to DO affinity of bull sharks monitored in the upper bay (Figure 8C & F).

## Discussion

Our data demonstrate the presence of juvenile bull sharks across a range of dynamic habitat conditions within Mobile Bay, Alabama. These findings add to a body of literature establishing the importance of Gulf of Mexico estuaries for bull sharks during this life stage [2], [20]–[22], and although not explicitly tested in the current study, suggests Mobile Bay could function as the northern most potential nursery area for bull sharks in the Gulf of Mexico. While we were unable to establish acoustic coverage across the entirety of Mobile Bay, we constructed an array designed to monitor bull shark presence along various ingress and egress points of the system and its freshwater sources. Rather than true “presence/absence” data, we suggest our findings provide a framework by which future, more specific, habitat use hypotheses can be tested.

Many of the Gulf of Mexico estuaries containing bull sharks have single fresh water point sources; however, Mobile Bay represents an interesting system that contains several sources of freshwater input, not all of which were used equally. For example, a disproportionate number of bull sharks were originally tagged along the western portion of Mobile Bay, at Dog River, yet the frequency of acoustic detections at this location was low. In contrast, no bull sharks were tagged in the upper bay, yet this area had the greatest proportion of detections throughout the CAAMP array. The most notable difference between the freshwater sources in the upper bay relative to the eastern and western bay is the magnitude of the riverine discharge. With an average discharge of 1800 m^3^sec^−1^, the upper portion of Mobile Bay receives the sixth largest river discharge in North America [33] and thus represents a unique habitat for young bull sharks. Comparing yearly detection differences in the upper bay in light of discharge data supports this idea. Mean freshwater discharge into Mobile Bay varies seasonally, with distinct wet and dry periods. Mean wet season (late winter, early spring) discharge into Mobile Bay is 2637 m^3^sec^−1^, compared to mean dry season (late summer/early fall) discharge of 802 m^3^sec^−1^ [34]. The upper bay experienced extreme discharge conditions between the two years we monitored bull sharks. Mean riverine discharge into Mobile Bay during the 2009 dry season was 2788 m^3^/sec (i.e. higher than typical wet season values), compared to 2010, where dry season discharge was 279 m^3^sec^−1^, three times lower than average conditions (USGS discharge data, waterdata.usgs.gov, accessed January 7, 2014). Bull sharks are known to alter dynamic habitat use in response to extreme weather events [8]; while we lack both the long term data and continuous acoustic coverage, the dramatic difference in riverine discharge between 2009 and 2010 offers a potential explanation for the difference in acoustic detections between 2009 and 2010 in the upper bay.

Discharge is clearly correlated to salinity, the most often cited parameter influencing the distribution of young bull sharks. In the eastern Gulf of Mexico, salinity was shown to be the most important physical parameter affecting bull shark movements, a relationship that was stronger for young-of-the-year individuals compared to animals older than 1 year [19]. Similarly, salinity was the only measured physical parameter shown to influence habitat use for immature bull sharks in the Indian River Lagoon [16]. The unique physical attributes of Mobile Bay make identifying the important hydrographic variables influencing habitat use more complex. Comparing two systems in Florida, where juvenile bull sharks have been well studied, to Mobile Bay Alabama illustrates this point. The Caloosahatchee River and the Indian River Lagoon are both classified as vertically homogeneous systems [35]. Both systems share similarly sized drainage basins (3,678 and 3,226 km^2^ for the Caloosahatchee and Indian River Lagoon, respectively) and receive similar average daily freshwater inputs (3,425 and 4,648 m^3^ day^−1^ for the Caloosahatchee and Indian River Lagoon, respectively). In comparison, Mobile Bay is classified as a moderately to highly stratified system, with a drainage area and average daily freshwater input thirty and forty times greater, respectively, than the Caloosahatchee and Indian River systems [35]. Our electivity analyses suggest when moderate salinities are available in the upper bay, as was the case in 2010, bull sharks were selecting for regions within that salinity range, similar to the patterns shown by bull sharks in the Caloosahatchee River [19] and along the Texas coast [15]. However, given the expansive nature of Mobile Bay, coupled with its dynamic hydrographic qualities, it is likely that factors in conjunction with salinity are influencing dynamic habitat use for young bull sharks.

The interactive nature of multiple predictive hydrographic parameters can be best seen when examining the unique relationship between salinity and dissolved oxygen in Mobile Bay. The affinity demonstrated by bull sharks in the lower bay for hypoxic waters was unexpected. In the Florida Everglades, dissolved oxygen has been shown to have the greatest influence on the probability of bull shark capture, with bull shark abundance greatest in areas where DO >3.5 mg L^−1^ [11]. We suggest the strong affinity shown for bull sharks in the lower bay for hypoxic waters is best considered with respect to other predictors. Shallow estuaries, typical of those juvenile bull sharks have been studied in previously, are generally well mixed [36]; however, despite being considered a shallow estuary, this is not the case with Mobile Bay. The moderate to high levels of vertical stratification in Mobile Bay set up a strong bottom to surface salinity gradient (ΔS), which is strongly correlated to bottom dissolved oxygen levels [37]. When ΔS is less than 2 psu, hypoxia in the shallow monitored regions of Mobile Bay is rare; conversely, when ΔS is greater than 4 psu, roughly 75% of the same area becomes hypoxic (here defined as DO <2 g m^−3^) [37]. Therefore, while periods of high freshwater discharge clearly lower salinity, they also establish a strong ΔS, which in turn can lead to wide-spread bottom hypoxia in the shallow regions of Mobile Bay. Our electivity data and others [19] demonstrate that while immature bull sharks are capable of inhabiting waters across a wide range of salinity, they tend to select waters from 7–11 psu. In Mobile Bay, these salinity regimes would be associated with a strong ΔS, and thus hypoxic conditions. This relationship offers a potential explanation for the apparent strong selection shown by young bull sharks in the lower bay in 2009 for hypoxic waters. The relationship between bull shark presence and hypoxia could also be explained by the limitations in our acoustic array. The electivity patterns we present need to be interpreted in light of our acoustic coverage, which we suggest provides “presence only” data. Interpreted in this respect, we may be simply detecting bull sharks as they exit Mobile Bay during hypoxic periods. Regardless of the mechanism, it seems clear that a combination of hydrographic variables influence the distribution of bull sharks monitored within Mobile Bay.

Temperature was the only main effect included in the final models for both lower and upper bays, in both 2009 and 2010. These findings complement previous studies investigating factors important in predicting the presence of young bull sharks. Analysis of the influence of salinity, temperature, depth, turbidity and DO on the distribution of juvenile bull sharks in the western Gulf of Mexico indicated salinity and temperature were the most influential factors, followed by freshwater inflow, turbidity and proximity to tidal inlets [15]. Interestingly, in Mobile Bay, the combination and strength of the factors that influence the distribution of young bull sharks varied over the two year period we examined. In the lower bay, the model fit the observations better in 2010, and with fewer parameters, all of which included temperature. Electivity patterns demonstrate that bull sharks appear to show preference for water greater than 30°C. We offer two potential explanations for these observations, the first being that bull sharks are simply selecting habitats with high water temperatures. Using multiple data sources, analysis of a thirty year data set from the Indian River Lagoon in Florida shows that the mean temperature of occurrence for juvenile bull sharks was 29.7°C [13], similar to the maximum electivity values shown for temperatures of 32 and 33°C for bull sharks in the lower bay in 2009 and 2010, respectively. In addition, acoustic tracking of an individual bull shark suggests long-term fidelity to thermal effluents in the Indian River Lagoon [16]. However, it could be that water temperatures in excess of 30°C represent a thermal maximum, and that our array detects the ensuing bull shark emigration from Mobile Bay, as suggested previously with dissolved oxygen.

While the interaction of hydrographic factors offers an explanation for the observed distribution of bull sharks in Mobile Bay, other factors not considered in this study, including the potential for increased prey availability and/or reduced mortality, are equally plausible. Several studies suggest that bull shark preference for freshwater is clearly not due to physiological constraints, and may be the result of increased resource abundance or decreased predation [38], [39]. Using long-term acoustic monitoring data, Heupel and Simpfendorfer [7] demonstrated that young bull sharks in the Caloosahatchee River Estuary suffered lower mortality rates relative to similarly sized tiger (*Galeocerdo cuvier*), school (*Galeorhinus galeus*), blacktip (*Carcharhinus limbatus*) and lemon (*Negaprion brevirostris*) sharks which occupy polyhaline (18–30 psu) environments. Further investigation is required to understand if either or both of these explanations (increased resource availability, decreased mortality) interact with hydrographic factors to influence habitat use throughout Mobile Bay.

Given the unique osmoregulatory capability of bull sharks, and their subsequent propensity to occupy estuarine habitats early in their ontogeny, this species may be especially susceptible to anthropogenic alterations to coastal ecosystems. In the Indian River Lagoon, bull sharks show affinity for urbanized habitats, a trend that can be partially explained by an affinity to thermal outfall from powerplants [16]. The ability of bull sharks to occupy these altered habitats may also provide population level benefits; decreased mortality has been shown for young bull sharks in highly urbanized areas in the Caloosahatchee River [7]. With respect to local-scale habitat selection, this suggests bull sharks, in this region and during this life stage, show a moderate degree of habitat specialization and are thereby sensitive to environmental fluctuation [40]. That said, neonate bull sharks monitored with acoustic tags along the East coast of Australia showed a preference for natural compared to artificial habitat [41], suggesting spatial variation in the degree of habitat specialization exhibited in this species. Combined, our data suggest that future studies coupling telemetry and hydrographic variables should, when possible, consider the interactions of multiple environmental parameters when identifying the dynamic variables explaining the spatial distribution of coastal shark species.
